# Analysis of retrospective natural history data collected from patients with SYNGAP1-related disorders: a preliminary examination of the Citizen database

**DOI:** 10.1186/s13023-025-03918-7

**Published:** 2025-07-27

**Authors:** Matthew R. Scott, Albert Misko, Yang Liu, Oleksandr Sverdlov

**Affiliations:** 1 Department of Biostatistics, School of Public Health, Boston University, Boston, MA USA; 2 Novartis Institutes for Biomedical Research, Cambridge, MA USA; 3 Novartis Pharmaceuticals Corporation, East Hanover, NJ USA

## Abstract

**Background:**

*SYNGAP1*-related disorder (SRD) is a rare neurodevelopmental disorder caused by genetic variants. A major challenge is the characterization of SRD, which requires assessment of several outcomes. We considered natural history data from the Citizen database on 65 patients with SRD in eight data domains: demographics, genetics, growth parameters, standardized clinical scales, developmental skills, neurological examinations, hospitalizations, and seizures. Exploratory analysis tools such as visualizations, summary statistics, and non-parametric statistical modeling were utilized.

**Results:**

Age at SRD diagnosis (median [IQR] = 3 [2, 5] years; [min, max] = [1, 17] years) was similar by sex. No evidence of a high frequency allele change in *SYNGAP1* was found, indicating no dominant variant in this patient population. Growth parameters of SRD children appeared normal in terms of height, weight, and head circumference. Developmental data were indicative of delayed development and language reversion. Standardized assessment data were largely sparse. Neurological exam data demonstrated ataxia and muscle tone issues. Hospitalization data highlighted substantial healthcare burden, largely due to seizures; absence, atonic, and myoclonic seizures were the most common types.

**Conclusion:**

Citizen data provide important insights into the natural course of SRD. Our findings not only provide utility in clinical practice of SRD but also contribute valuable insights to guide the development of SRD clinical trials. Limitations to our analysis include sparsity of standardized clinical scales data, crude statistical methodology, and bias induced by patients with older ages of diagnoses.

**Supplementary Information:**

The online version contains supplementary material available at 10.1186/s13023-025-03918-7.

## Introduction

Rare neurodevelopmental disorders (RNDDs) are neurological conditions caused by genetic variants that have substantial societal and economic impact. Variants of *SYNGAP1* lead to an imbalance of synaptic Ras guanosine triphosphatase (GTP)-activating protein 1, resulting in several neurological issues [2, 6]. Heterozygous deficiency of *SYNGAP1* is typically associated with non-syndromic intellectual disability (NSID), autism spectrum disorder (ASD), and epileptic encephalopathy [3, 4, 7, 8, 10, 15].

Studies have started to disentangle the natural history of *SYNGAP1*-related disorder (SRD) and identify outcomes for clinical trials of SRD. For instance, NSID is present in all SRD patients. Epilepsy is present in approximately 84.6% patients in [8] and 98% in Vlaskamp et al, 2018, with a portion of these patients even exhibiting pharmacoresistant epilepsy [9]. Delayed developmental skills are prominent in domains such as neuromotor or language, even before the onset of seizures [19].

Since the initial discovery of *SYNGAP1* and its association to NSID, epilepsy, and ASD in 2009, natural history studies of SRD have been published over the last decade, with studies such as Vlaskamp et al. 2018 (*n* = 57 SRD patients) and [17] (*n* = 147 SRD patients). Gene therapy programs have recently launched (e.g., GEN 2023 [5]), thus further characterization of SRD is needed for trial design as it requires assessment of several potential outcomes. This work aims to bridge the gap by running a preliminary investigation on 65 patients with SRD from the Citizen database. We utilized this dataset to investigate the natural history of SRD and inform the design of clinical trials in SRD.

## Methods

### Citizen data

This work analyzed 8 of 17 datasets provided by Citizen Health (Citizen, https://www.Citizen.com/syngap1) in 2021: demographics, genetics, growth parameters, standardized clinical scales, developmental skills, neurological exam findings, hospitalizations, and seizures. The Citizen database is a United States-based digital health platform that allows individuals with rare neurodevelopmental disorders to contribute their de-identified medical records for research. Participation is voluntary and initiated by self-enrollment, though many individuals in this study were recruited through collaborations with patient advocacy groups such as the SynGAP Research Fund. Participants included in this study had genetically confirmed pathogenic or likely pathogenic *SYNGAP1* variants identified through routine clinical testing. Ethnicity data are not available in this database.

**Fig. 1:**
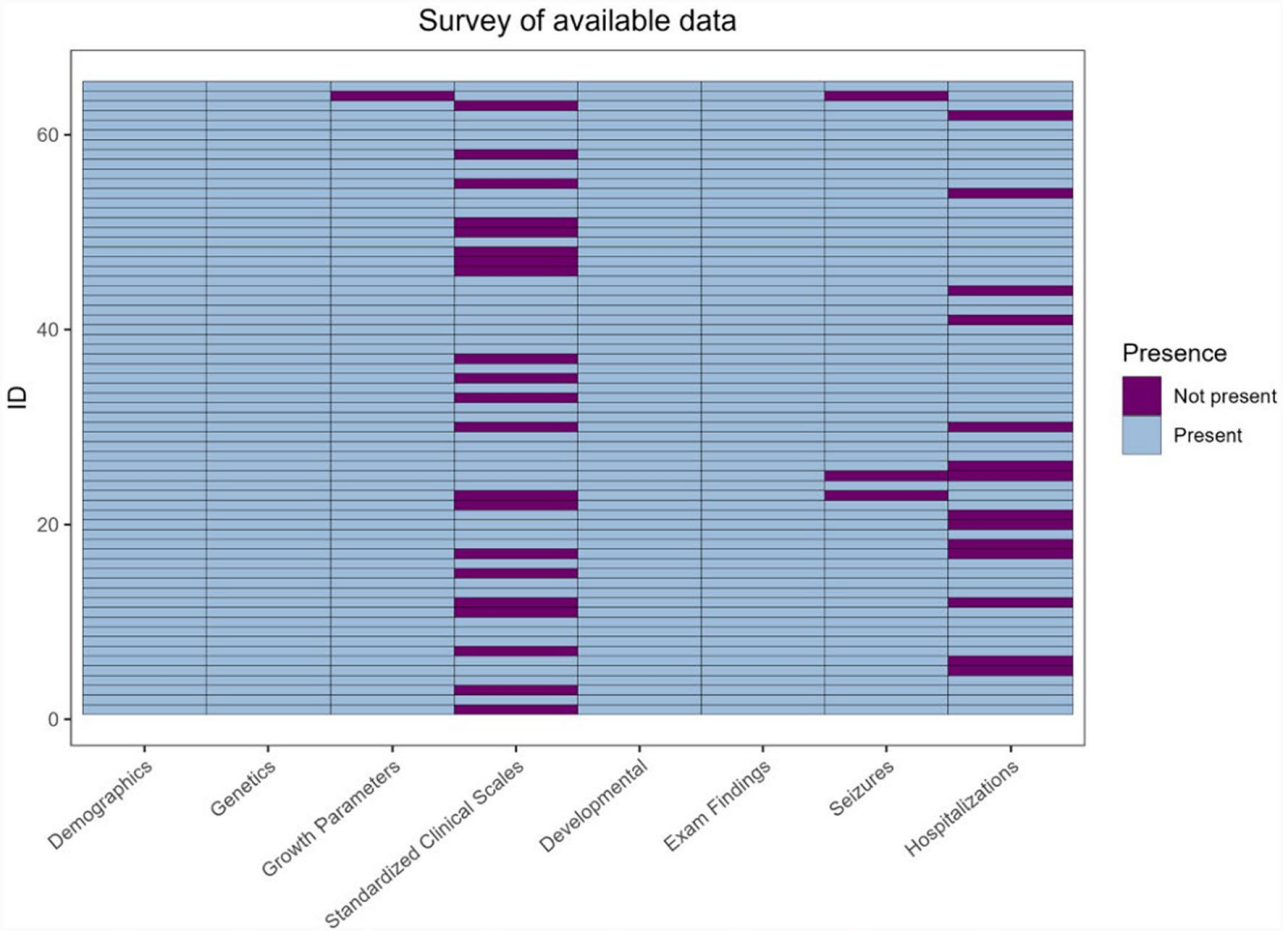
Availability of patient data in each Citizen dataset

65 patients with SRD were present, each with varying records across the different datasets. The presence of each patient’s records is reported in Fig. 1. Within each dataset, we aimed to determine if there were sufficient data for meaningful longitudinal or cross-sectional analyses. Most patients had records in each dataset, but the standardized clinical scales file was sparse due to a lack of harmonization between the scales (Supplemental Table 1). For example, the Peabody Developmental Motor Scales 2nd Edition was the highest frequency scale, yet it only had 19 patient records. Moreover, the highest frequency subdomain in this scale was the Grasping Standard Score, which had data for only 10 patient records (7 cross-sectional and 3 longitudinal). Though pertinent to understanding the natural history of SRD, standardized clinical scales data were not utilized due to this sparsity.

**Table 1:** Frequency tables of alleles, amino acid changes, inheritance, and interpretation of pathogenicity in the *SYNGAP1* gene (NM_006772.3 transcript)

Genetic allele	Amino acid change	Variant type	Interpretation	N (%)
chr16:33,451,137–33,543,925 × 1		CNV	Pathogenic	1 (1.5)
chr6:33,265,327–33,600,853 × 1		CNV	Pathogenic	1 (1.5)
chr6:33,399,937–33,400,584 × 1		CNV	Pathogenic	1 (1.5)
Gain (Exon 3)		CNV	Likely pathogenic	1 (1.5)
c.3416dupA	p.Thr1140Aspfs*13	Duplication, Frameshift	Pathogenic	1 (1.5)
c.698_699dupGT	p.Arg234Valfs*18	Duplication, Frameshift	Pathogenic	1 (1.5)
c.1760_1792del33	p.Arg587_Leu598delinsIle	Indel, Complex	Pathogenic	1 (1.5)
c.1022_1030delinsTA	p.Gly341Valfs*4	Indel, Frameshift	Pathogenic	1 (1.5)
c.1154_1161del	p.Ser385Trpfs*31	Indel, Frameshift	Pathogenic	1 (1.5)
c.1167_1168delAG	p.Gly391Glnfs*27	Indel, Frameshift	Pathogenic	1 (1.5)
c.1167delA	p.Gly391Alafs*12	Indel, Frameshift	Pathogenic	1 (1.5)
c.1463delC	p.Thr488Serfs*7	Indel, Frameshift	Pathogenic	1 (1.5)
c.2293delA	p.Ser765Alafs*44	Indel, Frameshift	Pathogenic	1 (1.5)
c.2561_2577del	p.Leu840fs	Indel, Frameshift	Pathogenic	1 (1.5)
c.2843del	p.Gly948Alafs*129	Indel, Frameshift	Pathogenic	1 (1.5)
c.3233_3236del	p.Val1078Alafs*51	Indel, Frameshift	Pathogenic	1 (1.5)
c.333del	p.Lys114Serfs*20	Indel, Frameshift	Pathogenic	1 (1.5)
c.333delA	p.Lys114Serfs*20	Indel, Frameshift	Pathogenic	1 (1.5)
c.404_411delGACGGCTAinsT	p.Arg135Leufs*37	Indel, Frameshift	Likely pathogenic	1 (1.5)
c.410delT	p.Leu137Glnfs*37	Indel, Frameshift	Pathogenic	1 (1.5)
c.411_418delAAAAAGCT	p.Lys138Hisfs*11	Indel, Frameshift	Pathogenic	1 (1.5)
c.535delG	p.Glu179Serfs*9	Indel, Frameshift	Pathogenic	1 (1.5)
c.654_655del	p.Phe218Leufs*3	Indel, Frameshift	Pathogenic	1 (1.5)
c.745del	p.Ala249Glnfs*2	Indel, Frameshift	Pathogenic	1 (1.5)
c.768_770delCAGinsAA	p.Asn256Lysfs*5	Indel, Frameshift	Pathogenic	1 (1.5)
c.878delG	p.Arg293Profs*54	Indel, Frameshift	Pathogenic	1 (1.5)
c.3452_3453del	p.Ser1151Ter	Indel, Nonsense	Pathogenic	1 (1.5)
c.190-2A > G		Splice-site	Pathogenic	1 (1.5)
c.1913 + 1G > A		Splice-site	Pathogenic	1 (1.5)
c.3583-6G > A		Splice-site	Pathogenic	1 (1.5)
c.3583-9G > A		Splice-site	Pathogenic	1 (1.5)
c.388-3C > G		Splice-site	Likely pathogenic	1 (1.5)
c.190-15_206delins28		Splice-site, Indel	Pathogenic	1 (1.5)
c.1030G > A	p.Gly344Ser	Substitution, Missense	Likely pathogenic	1 (1.5)
c.1292 T > C	p.Leu431Pro	Substitution, Missense	Likely pathogenic	1 (1.5)
c.1403 T > A	p.Met468Lys	Substitution, Missense	Likely pathogenic	1 (1.5)
c.1797C > G	p.Cys599Trp	Substitution, Missense	Likely pathogenic	1 (1.5)
c.1889 T > A	p.lle630Asn	Substitution, Missense	Likely pathogenic	1 (1.5)
c.1946 T > G	p.Met649Arg	Substitution, Missense	Likely pathogenic	1 (1.5)
c.662A > T	p.Glu221Val	Substitution, Missense	Likely pathogenic	1 (1.5)
c.851 T > C	p.Leu284Pro	Substitution, Missense	Likely pathogenic	1 (1.5)
c.490C > T	p.Arg164Ter	Substitution, Nonsense	Pathogenic	4 (6.2)
c.3718C > T	p.Arg1240Ter	Substitution, Nonsense	Pathogenic	3 (4.6)
c.1861C > T	p.Arg621Ter	Substitution, Nonsense	Pathogenic	2 (3.1)
c.403C > T	p.Arg135Ter	Substitution, Nonsense	Pathogenic	2 (3.1)
c.1284 T > A	p.Tyr428Ter	Substitution, Nonsense	Pathogenic	1 (1.5)
c.1507C > T	p.Gln503Ter	Substitution, Nonsense	Pathogenic	1 (1.5)
c.1735C > T	p.Arg579Ter	Substitution, Nonsense	Pathogenic	1 (1.5)
c.2059C > T	p.Arg687Ter	Substitution, Nonsense	Pathogenic	1 (1.5)
c.2104C > T	p.Gln702Ter	Substitution, Nonsense	Pathogenic	1 (1.5)
c.2755C > T	p.Gln919Ter	Substitution, Nonsense	Pathogenic	1 (1.5)
c.2899C > T	p.Arg967Ter	Substitution, Nonsense	Pathogenic	1 (1.5)
c.2946 T > A	p.Tyr982Ter	Substitution, Nonsense	Pathogenic	1 (1.5)
c.3277C > T	p.Gln1093Ter	Substitution, Nonsense	Pathogenic	1 (1.5)
c.3316C > T	p.Gln1106Ter	Substitution, Nonsense	Pathogenic	1 (1.5)
c.3553A > T	p.Lys1185Ter	Substitution, Nonsense	Pathogenic	1 (1.5)
c.984C > G	p.Tyr328Ter	Substitution, Nonsense	Likely pathogenic	1 (1.5)
c.2115G > A	p.Lys705 =	Substitution, Synonymous	Pathogenic	1 (1.5)
Total				65 (100)

### Analysis

Our approach was largely exploratory; visualization techniques, summary statistics, and non-parametric modeling were frequently used. Non-parametric statistical modeling included generalized additive models (GAMs), Mann–Whitney U tests, and Kaplan–Meier analyses. Visualizations included histograms, grid/panel plots, and scatter plots. Summary statistics included measures of frequency, central tendency (mean and median), and variability (IQR, [min, max], SD). Frequency was calculated by summing across all observations, as well as across patients, where a patient was counted once if they had at least one finding over any of their follow-up records. All analyses were performed using R v4.2.2. The variables and analyses in each dataset were as follows:

I. Demographics data consisted of age at diagnosis as well as sex. Histograms and descriptive statistics of age at diagnosis were created across the full sample and stratified by sex. A Mann–Whitney U test was used to assess if age at diagnosis differed by sex. A smoothed GAM plot was used to visualize how age at diagnosis changes as a function of birth year.
II. Genetics data contained allele changes, amino acid changes, interpretation of pathogenicity, and inheritance. In *SYNGAP1*, variant allele change, amino acid change, and interpretations of pathogenicity were tabulated concurrently. Frequency of inheritance is additionally reported. Interpretations of pathogenicity were determined by the variant classification criteria of the American College of Medical Genetic and Genomics, and the Association for Molecular Pathology [13].
III. Growth parameters consisted of longitudinal body weight, body height, and head circumference. Sufficient data was present for each patient to perform a meaningful longitudinal analysis (Supplemental Table 2). GAMs were used to construct growth curves of each parameter over time (age) against published Center for Disease Control or World Health Organization 50th percentile norms stratified by sex and age (male or female; 0–2 years or 3 + years).
IV. Developmental data comprised four domains (academic, language, fine motor, and gross motor) with specific skills in each domain (e.g., ability to babble in the language domain). Frequencies of skills in each domain are reported in Supplemental Table 3. Skills that were clinically relevant to neurologists, had sufficient frequency, and did not have ambiguous definitions were used in analyses. The academic domain was not analyzed as it did not meet these criteria. Longitudinal ability or inability status was visualized in 6-month intervals for each patient using grid plots. Kaplan–Meier cumulative density function curves were used to illustrate ages of skill attainment. Median ages of skill attainment were estimated.
V. Neurological exam findings contained five domains (muscle tone, gait, cranial nerves, coordination, and sensation) with specific records in each domain (e.g., hypotonia in the muscle tone domain). Frequency of records by domain are reported. To visualize issues over time in each domain, a panel plot was used to show abnormal and normal records in 6-month intervals for each patient.
VI. Hospitalization data consisted of age of hospitalization as well as the cause of hospitalization. Frequency of hospitalization causes were tabulated to illustrate factors driving healthcare and patient burden. Grid plots were used to visualize the number of hospitalizations at each year of age.
VII. Frequency of seizure type was assessed to identify common manifestations of epilepsy.

**Table 2:** Seizure frequency across patients

Type of seizure	N (%) Patients
*Total Patients*	*62 (100.0)*
Absence seizure	33 (53.2)
Atonic seizures	31 (50)
Atypical absence seizure	18 (29)
Myoclonic seizure	12 (19.4)
Tonic–clonic seizure	12 (19.4)
Eyelid myoclonia	8 (12.9)
Focal seizure	4 (6.5)
Tonic seizure	4 (6.5)
Absence seizure with atonic components	3 (4.8)
Eyelid myoclonus with absences	3 (4.8)
Myoclonic-atonic seizure	2 (3.2)
Subclinical seizure	2 (3.2)
Clonic seizure	1 (1.6)
Complex partial seizure with impairment of consciousness	1 (1.6)
Generalized tonic–clonic seizure	1 (1.6)
Myoclonic absence seizure	1 (1.6)

## Results

### Demographics

**Fig. 2:**
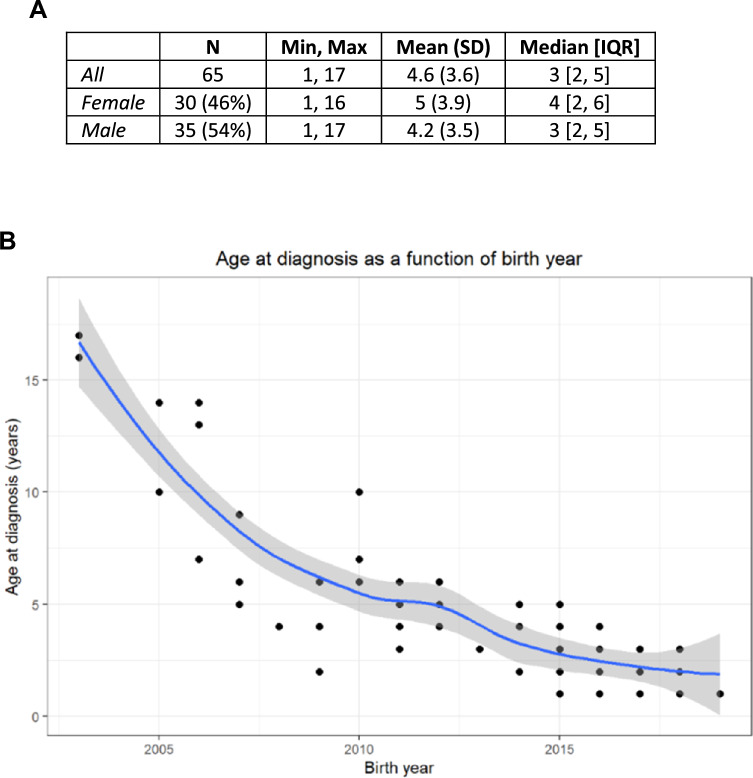
Age at diagnosis. **A** Age of diagnosis (years) in the full sample and by sex. **B** Age of diagnosis as a function of birth year

The sample consisted of 30 (46%) females and 35 males (54%). Descriptive statistics for age of diagnosis and a smoothed scatterplots of age of diagnosis as a function of birthday are reported in Fig. 2. Age of diagnosis did not differ by sex (U = 598.5, *p* = 0.33). Age at diagnosis decreased as birth year increases.

### Genetics

Allele change, amino acid change, and variant type frequencies are reported in Table 1. The c.490C > T allele and p.Arg164Ter amino acid change had the highest frequency (*n* = 4); no dominant allele or amino acid change was present in this patient population. Indel (*n* = 21) and substitution (*n* = 32) variant types were prominent, and most patients had pathogenic interpretations (*n* = 53). Inheritance was predominantly de novo (*n* = 38), with only n = 1 maternal mosaic and *n* = 26 missing.

### Growth parameters

**Fig. 3:**
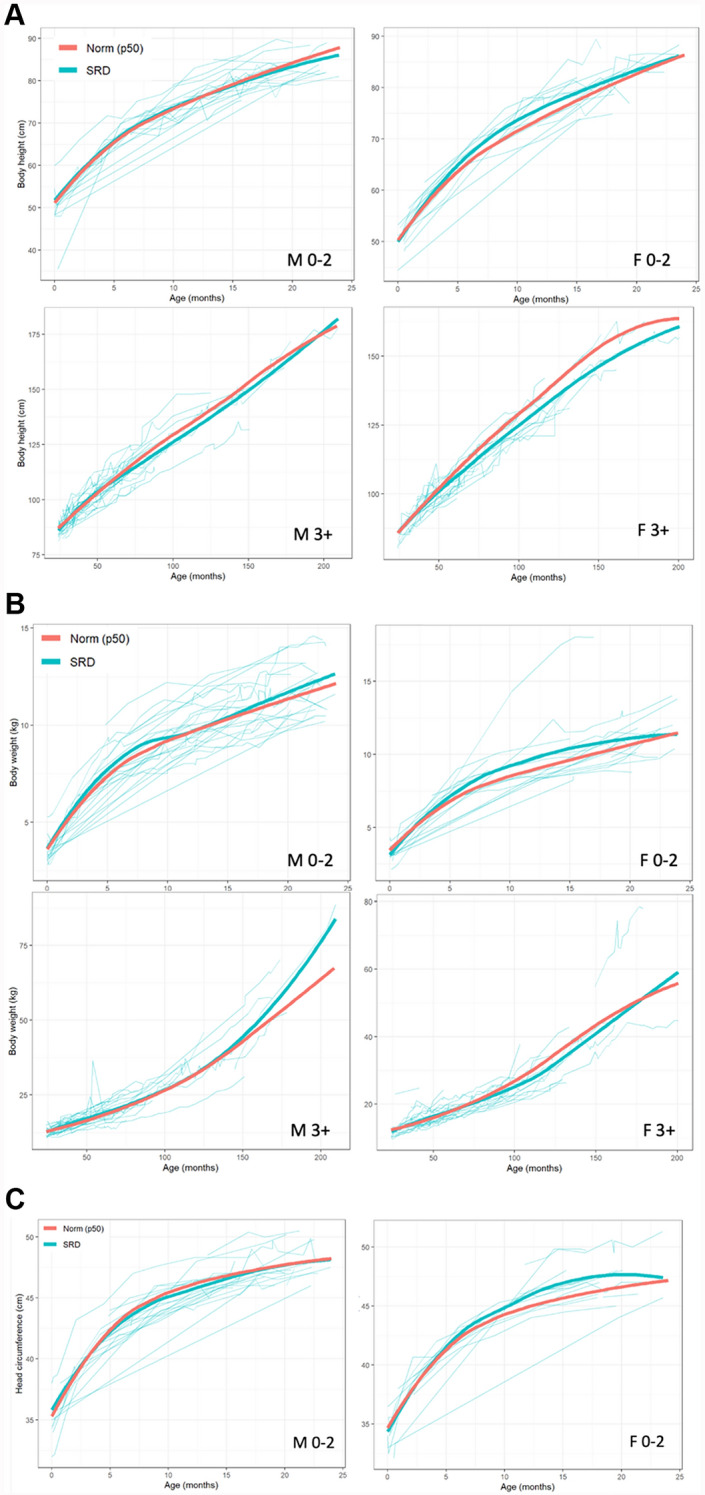
Growth curves for SRD patients (teal) compared to 50th percentile norms (red). **A** Height (upper left: males 0–2 years; lower left: males 3 + years; upper right: females 0–2 years; lower right: females 3 +). **B** Weight (upper left: males 0–2 years; lower left: males 3 + years; upper right: females 0–2 years; lower right: females 3 +). **C** Head circumference (left: males 0–2 years; right: females 0–2 years)

Growth parameters of SRD patients and 50th percentile norms were plotted over time by age and sex. Height, weight, and head circumference curves are reported in Fig. 3. Growth curves were typical across the three parameters when compared to established norms. However, minor deviations were present. For example, the 3 + females had slightly lower height at older ages compared to the norm, but this was likely due to a reduced sample size in stratification.

### Developmental skills

**Fig. 4:**
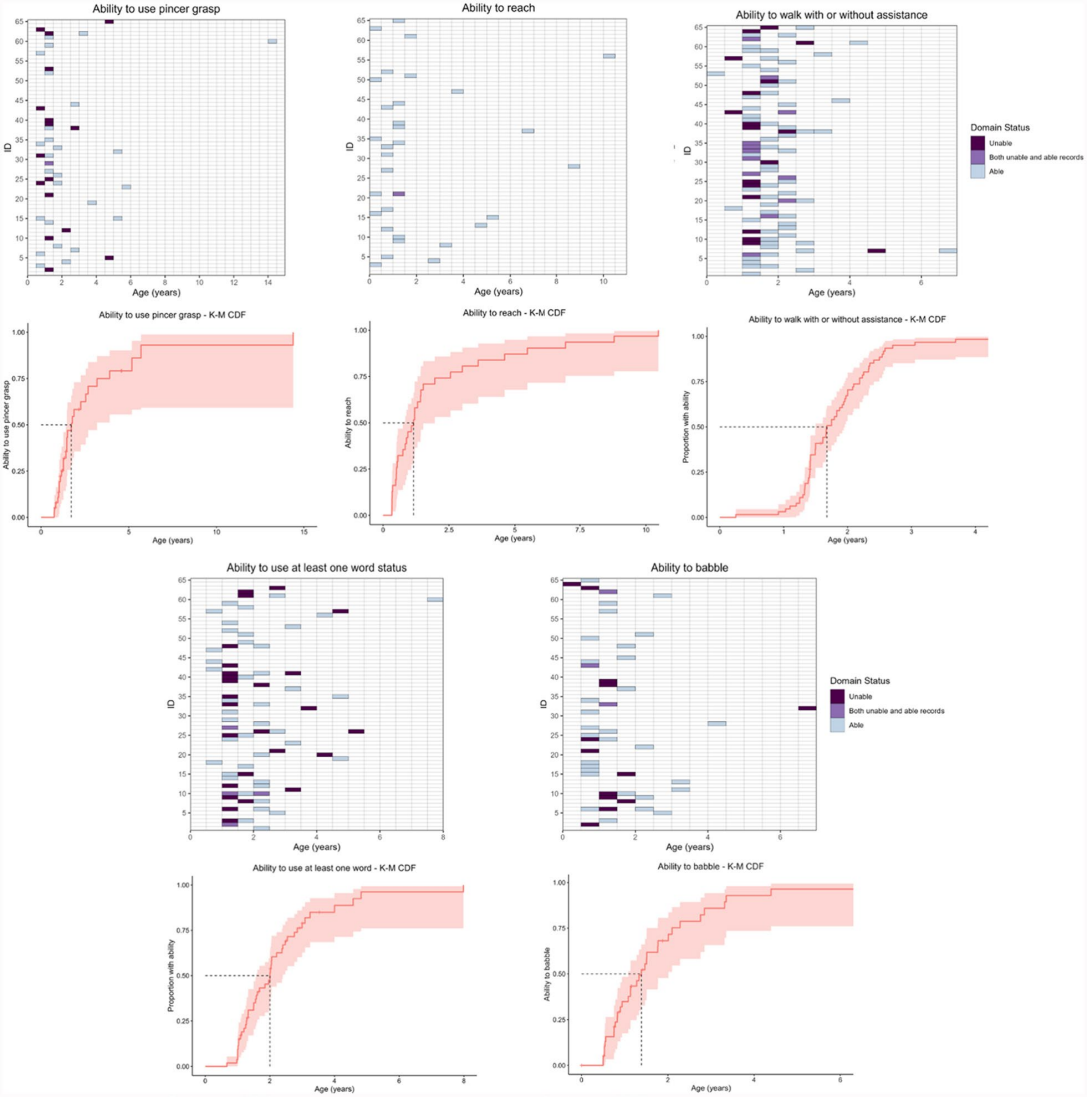
Developmental skills across domains of language, fine motor, and gross motor. Grid plots are shown in 6-month intervals. Dark colors indicate inability to perform a skill while light colors indicate ability to perform a skill. The gradient in-between indicates mixed reports in the 6-month interval. Kaplan–Meier survival plots for skill attainment are also displayed. Dashed lines indicate the median age of skill attainment

Language, fine motor, and gross motor domains were analyzed. Grid plots and Kaplan–Meier cumulative density plot estimates are reported in Fig. 4. For language, the ability to use at least one word and ability to babble skills were used. Ability to use at least one word had n = 64, events = 62, 6 patients reverted in the skill, and median [95% CI] = 1.68 [1.5, 1.96] years. Ability to babble had n = 40, events = 32, 2 patients reverted, and median [95% CI] = 1.39 [1.08, 2.01] years. For gross motor, the ability to walk with or without assistance skill was used; it had n = 64, events = 62, 1 patient reverted, and median [95% CI] = 1.68 [1.5, 1.96] years. For fine motor, the ability to use pincer grasp and ability to grasp skills were used. Ability to use pincer grasp had n = 38, events = 26, 1 patient reverted, and median [95% CI] = 1.72 [1.42, 3.19] years. Ability to reach had n = 31, events = 31, 1 patient reverted, and median [95% CI] = 1.16 [0.75, 1.96] years. Overall, estimated medians were indicative of delayed development, and skill regression was observed in the language domain.

### Neurological exam findings

**Fig. 5:**
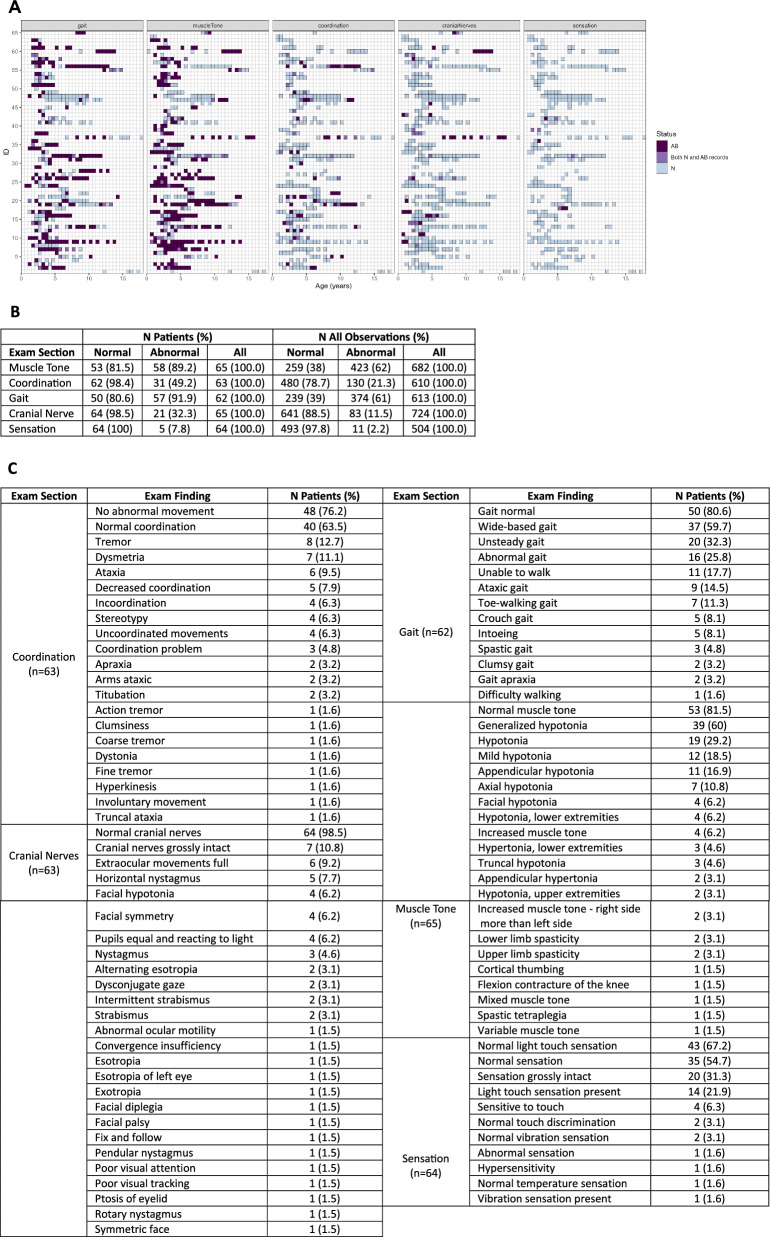
Neurological exam abnormalities over time. **A** Panel plot of abnormal exam findings over time for each patient (rows) in 6-month intervals. Darker colors indicate abnormal findings, light colors indicate normal findings, and the gradient indicates mixed reports during the 6-month interval. **B** Frequency of abnormalities across patients and all observations. **C** Frequencies of neurological exam findings in each domain across patients

A panel plot and frequency table highlighting neurological exam abnormalities over time are reported in Fig. 5. Gait and muscle tone were the most frequent abnormalities, with approximately 90% of patients experiencing at least one issue in these domains. The highest frequency concern in the muscle tone domain was hypotonia of varying degrees. In the gait domain, wide-based, unsteady, and/or abnormal gait as well as an inability to walk were common. For coordination, ataxia and tremors were present. Nystagmus was the most frequent problem in the cranial nerve domain, but this was not commonly or not frequently recorded. Only five patients had an abnormal sensation record.

### Hospitalizations

**Fig. 6:**
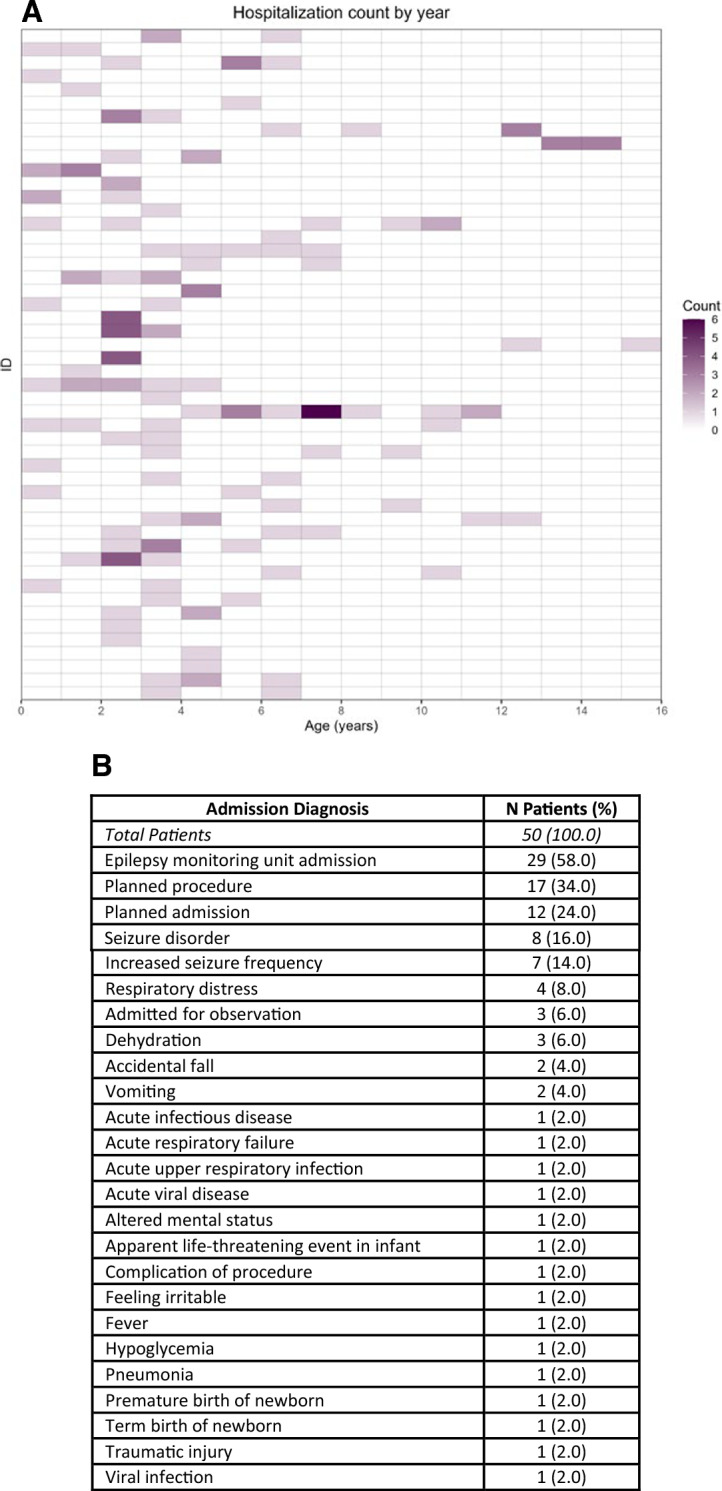
Hospitalization frequency and causes. **A** Grid plot showing the number of all-cause hospitalizations per year in each patient (rows), with darker colors indicating higher admission frequency. **B** Hospital admission diagnosis frequency across patients

51 of the 65 SRD patients had hospitalizations records. A grid plot of all cause hospitalization frequency by age is reported in Fig. 6, showing frequent hospitalizations at younger ages. Seizures/epilepsy and planned procedures/admissions were the most common causes of hospital admissions for SRD patients. A breakdown of these causes is reported in Fig. 6.

### Seizures

Frequency of seizure types are reported in Table 2. Absence seizures were the most prevalent, followed by atonic and myoclonic seizures.

## Discussion

This work employed Citizen data to characterize the nuanced and complex natural history of the SRD. Demographics, genetics, growth parameters, clinical scales, developmental skills, neurological exam findings, and hospitalizations data were analyzed. We first assessed completeness of data and found the standardized clinical scales data to be sparse. The remaining files were investigated using exploratory analysis methods.

Age at diagnosis did not differ by sex, indicating no apparent sex difference in the clinical manifestations of SRD. We observed that age at diagnosis decreased as a function of time, likely due to clinical sequencing becoming more commonplace in recent years [14]. For genetics data, no high frequency allele or amino acid change was present, indicating no dominant variant in this patient population. As expected, interpretations were pathogenic and inheritance was de novo. Growth curves of height, weight, and head circumference were compared to 50th percentile norms by age and sex. No major deviations in SRD patients were observed in any of the three growth parameters, indicating typical growth patterns in SRD patients. This contrasts with Lo Barco et al., who reported low auxological parameters in male SRD patients.

Development delay is common in SRD patients [7, 8, 12, 14, 15]. Citizen developmental data provided an extensive array of skills that were useful for investigating and scrutinizing the intricacies of this phenomenon. Across all five skills analyzed, median ages of skill attainment were slightly higher than those of a normal population. For example, the median age of walking with or without assistance was 1.68 years. However, in a normal population a child is expected to be walking with assistance at 9 months or alone at 1 year [18]. Another example is the ability to speak at least one word. The median age in SRD patients was 2 years, but in a normal population it is approximately 7–12 months [1]. Therefore, our findings indicate delayed development in SRD patients. Language regression was found in 6 of the 53 patients for the ability to use at least one word. This is a common feature of ASD [11], so regression in this skill may be a manifestation of ASD in SRD patients.

For neurological exam data, muscle tone and gait showed a higher frequency of abnormal reports compared to other exam metrics, consistent with previous reports of hypotonia and ataxia [15]. While more complex modeling methodologies are needed to fully elucidate longitudinal patterns of these abnormalities, the panel plot showed these issues remaining consistent over multiple follow-up visits. We observed recurrent hospitalizations at younger ages, highlighting the burden of hospitalizations on SRD patients. Hospitalization frequency decreased at later ages, possibly due to patients attaining stable treatment or due to a lack of follow-up records. Consistent with previous literature [7, 8, 15, 16], absence, atonic, and myoclonic seizures were dominant in the seizure data.

There are several key limitations to consider. While this sample was large compared to other studies of SRD, records were sparse in key domains pivotal to understanding the natural course of SRD. For example, standardized clinical scales may provide important insight into the progression of intellectual disability, but it was insufficient due to a lack of harmonized testing. Sleeping problems have also been reported in SRD patients [8, 10, 15], but sleep-related difficulties or insomnia data were outside the scope of our analysis. Recent work has investigated genotype–phenotype correlations in SRD. For example, [17] used Citizen data to show that individuals with variants in the SH3 domain were less likely to have epilepsy, while those with mutations in exons 1 through 4 tended to exhibit milder language delays. Future research should explore these correlations to better understand causes of heterogeneous SRD phenotypes.

More elaborate modeling methodologies may be explored in future work. For example, GAMs were utilized for their ability to model complex non-linear patterns with minimal assumptions. However, future methods may more explicitly account for within-patient variation or unbalanced follow-up records. Kaplan–Meier methods were used to estimate median age of developmental skill attainment. However, medical records only noted if a skill was or was not present, and a patient may have developed a skill earlier than their medical record indicates. Analyses accounting for missing data could be incorporated in future work to validate our findings. This work only analyzed univariate or bivariate associations. Given the complexity and multifaceted manifestation of SRD, analyses should examine multivariate associations and interactions to create more complete models of disease progression and staging. Lastly, there are additional Citizen datasets such as medications or adverse events that should be investigated in future work.

## Conclusion

Citizen data revealed genetic variability in the *SYNGAP1* gene, normal growth patterns, developmental delays and language reversion, gait and muscle issues, hospitalization burden in SRD patients, and seizure types in the largest natural history sample to date. To further disentangle the natural history of SRD, additional analyses should be performed with Citizen data and supplemented with data that can account for sparsity in certain domains.

## Supplementary Information

Supplementary file 1.

## Data Availability

The data that support the findings of this study are available from Citizen Health (https://www.citizen.com/syngap1/) but restrictions apply to the availability of these data, which were used under license for the current study, and so are not publicly available. Data are however available from the authors upon reasonable request and with permission of Citizen.

## References

[1] American Speech-Language-Hearing Association. “Communication Milestones: Birth to 1 Year.” www.asha.org, www.asha.org/public/developmental-milestones/communication-milestones-birth-to-1-year/.

[2] Berryer MH, Hamdan FF, Klitten LL, et al. Mutations in SYNGAP1 cause intellectual disability, autism, and a specific form of epilepsy by inducing haploinsufficiency. Hum Mutat. 2013;34(2):385–94. 10.1002/humu.22248. 23161826 10.1002/humu.22248

[3] Clement JP, Aceti M, Creson TK, et al. Pathogenic SYNGAP1 mutations impair cognitive development by disrupting maturation of dendritic spine synapses. Cell. 2012;151(4):709–23. 10.1016/j.cell.2012.08.045. 23141534 10.1016/j.cell.2012.08.045 PMC3500766

[4] Carvill GL, Heavin SB, Yendle SC, et al. Targeted resequencing in epileptic encephalopathies identifies de novo mutations in CHD2 and SYNGAP1. Nat Genet. 2013;45(7):825–30. 10.1038/ng.2646. 23708187 10.1038/ng.2646 PMC3704157

[5] Genetic Engineering & Biotechnology News (GEN). (2023). Gene therapy team wins grant to further research on rare SYNGAP-1 genetic disorder. https://www.genengnews.com/news/gene-therapy-team-wins-grant-to-further-research-on-rare-syngap-1-genetic-disorder/

[6] Hamdan FF, Gauthier J, Spiegelman D, et al. Mutations in SYNGAP1 in autosomal nonsyndromic mental retardation. N Engl J Med. 2009;360(6):599–605. 10.1056/NEJMoa0805392. 19196676 10.1056/NEJMoa0805392 PMC2925262

[7] Jimenez-Gomez A, Niu S, Andujar-Perez F, et al. Phenotypic characterization of individuals with SYNGAP1 pathogenic variants reveals a potential correlation between posterior dominant rhythm and developmental progression. J Neurodev Disord. 2019;11(1): 18. 10.1186/s11689-019-9276-y. 31395010 10.1186/s11689-019-9276-y PMC6688356

[8] Lo Barco T, De Gaetano L, Santangelo E, et al. SYNGAP1-related developmental and epileptic encephalopathy: the impact on daily life. Epilepsy Behav. 2022;127: 108500. 10.1016/j.yebeh.2021.108500. 34954508 10.1016/j.yebeh.2021.108500

[9] Mignot C, von Stülpnagel C, Nava C, et al. Genetic and neurodevelopmental spectrum of SYNGAP1-associated intellectual disability and epilepsy. J Med Genet. 2016;53(8):511–22. 10.1136/jmedgenet-2015-103451. 26989088 10.1136/jmedgenet-2015-103451

[10] Parker MJ, Fryer AE, Shears DJ, et al. De novo, heterozygous, loss-of-function mutations in SYNGAP1 cause a syndromic form of intellectual disability. Am J Med Genet A. 2015;167A(10):2231–7. 10.1002/ajmg.a.37189. 26079862 10.1002/ajmg.a.37189 PMC4744742

[11] Pickles A, Wright N, Bedford R. Predictors of language regression and its association with subsequent communication development in children with autism. J Child Psychol Psychiatry. 2022;63(11):1243–51. 10.1111/jcpp.13565. 35098539 10.1111/jcpp.13565 PMC9786608

[12] Ribeiro-Constante J, Tristán-Noguero A, Martínez Calvo FF, et al. Developmental outcome of electroencephalographic findings in *SYNGAP1* encephalopathy. Front Cell Dev Biol. 2024;12: 1321282. 10.3389/fcell.2024.1321282. 38505260 10.3389/fcell.2024.1321282 PMC10948473

[13] Richards S, Aziz N, Bale S, et al. ACMG Laboratory Quality Assurance Committee. Standards and guidelines for the interpretation of sequence variants: a joint consensus recommendation of the American College of Medical Genetics and Genomics and the Association for Molecular Pathology. Genet Med. 2015; 17(5):405–24 10.1038/gim.2015.30 PMC4544753 25741868

[14] Rong M, Benke T, Zulfiqar Ali Q, Aledo-Serrano Á, Bayat A, Rossi A, Devinsky O, Qaiser F, Ali AS, Fasano A, Bassett AS, Andrade DM. Adult Phenotype of *SYNGAP1*-DEE. Neurol Genet. 2023;9(6): e200105. 10.1212/NXG.0000000000200105. 38045990 10.1212/NXG.0000000000200105 PMC10692795

[15] Vlaskamp DRM, Shaw BJ, Burgess R. SYNGAP1 encephalopathy: a distinctive generalized developmental and epileptic encephalopathy. Neurology. 2019;92(2):e96–107. 10.1212/WNL.0000000000006729. 30541864 10.1212/WNL.0000000000006729 PMC6340340

[16] von Stülpnagel C, Hartlieb T, Borggräfe I, et al. Chewing induced reflex seizures (“eating epilepsy”) and eye closure sensitivity as a common feature in pediatric patients with SYNGAP1 mutations: review of literature and report of 8 cases. Seizure. 2019;65:131–7. 10.1016/j.seizure.2018.12.020. 30685520 10.1016/j.seizure.2018.12.020

[17] Wiltrout K, Brimble E, Poduri A. Comprehensive phenotypes of patients with SYNGAP1-related disorder reveals high rates of epilepsy and autism. Epilepsia. 2024;65(5):1428–38. 10.1111/epi.17913. 38470175 10.1111/epi.17913 PMC12375243

[18] World Health Organization. “Motor Development Milestones.” www.who.int, www.who.int/tools/child-growth-standards/standards/motor-development-milestones.

[19] Zhang H, Yang L, Duan J, et al. Phenotypes in children with SYNGAP1 encephalopathy in China. Front Neurosci. 2021;15: 761473. 10.3389/fnins.2021.761473. 34924933 10.3389/fnins.2021.761473 PMC8678593

