# Supplementary material for: Analysis of retrospective natural history data collected from patients with SYNGAP1-related disorders: a preliminary examination of the Citizen database
Source: Orphanet J Rare Dis. 2025 Jul 27;20:379. doi: 10.1186/s13023-025-03918-7 (PMC12302738; doi:10.1186/s13023-025-03918-7)
Supplement: Supplementary file 1 [file 13023_2025_3918_MOESM1_ESM.docx]

**Supplemental Material**

*Analysis of retrospective natural history data collected from patients with SYNGAP1-related disorders: a preliminary examination of the Citizen database*

*Scott et al. 2025*

**Table 1.** Standardized clinical scale data availability

**Table 1.A.** Scale frequency

| **Scale** | **No records** | **1 record** | **2 records** | **3 or more records** |
| --- | --- | --- | --- | --- |
| PEABODY DEVELOPMENTAL MOTOR SCALES, 2ND EDITION | 25 | 12 | 5 | 2 |
| VINELAND ADAPTIVE BEHAVIOR SCALES | 28 | 11 | 5 | 0 |
| MULLEN SCALES OF EARLY LEARNING | 34 | 8 | 1 | 1 |
| CAPUTE SCALES | 34 | 7 | 1 | 2 |
| ADOS | 38 | 6 | 0 | 0 |
| BAYLEY SCALES OF INFANT DEVELOPMENT | 36 | 6 | 2 | 0 |
| GESELL DEVELOPMENTAL OBSERVATION REVISED | 37 | 6 | 0 | 1 |
| PLS-5 - PRESCHOOL LANGUAGE SCALE FIFTH EDITION | 36 | 6 | 1 | 1 |
| ABAS-3 - ADAPTIVE BEHAVIOR ASSESSMENT SYSTEM THIRD EDITION | 40 | 4 | 0 | 0 |
| BEERY-BUKTENICA TEST OF VISUAL MOTOR INTEGRATION | 40 | 4 | 0 | 0 |
| CARS2 - CHILDHOOD AUTISM RATING SCALE, 2ND EDITION | 40 | 4 | 0 | 0 |
| REEL-3 - RECEPTIVE EXPRESSIVE EMERGENT LANGUAGE TEST THIRD EDITION | 39 | 4 | 1 | 0 |
| DAYC-2 - DEVELOPMENTAL ASSESSMENT OF YOUNG CHILDREN SECOND EDITION | 40 | 3 | 1 | 0 |
| PLS-4 - PRESCHOOL LANGUAGE SCALE FOURTH EDITION | 39 | 3 | 2 | 0 |
| WECHSLER PRE-SCHOOL AND PRIMARY SCALE OF INTELLIGENCE | 39 | 3 | 2 | 0 |
| BAYLEY SCALE OF INFANT DEVELOPMENT | 42 | 2 | 0 | 0 |
| BEHAVIOUR ASSESSMENT SYSTEM FOR CHILDREN | 42 | 2 | 0 | 0 |
| STANFORD BINET INTELLIGENCE SCALE | 42 | 2 | 0 | 0 |
| AUTISM DIAGNOSTIC OBSERVATION SCHEDULE (ADOS) | 43 | 1 | 0 | 0 |
| BATELLE DEVELOPMENTAL INVENTORY | 43 | 1 | 0 | 0 |
| BATTELLE DEVELOPMENTAL INVENTORY SCREENING TOOL | 43 | 1 | 0 | 0 |
| BRACKEN BASIC CONCEPT SCALE EXPRESSIVE | 43 | 1 | 0 | 0 |
| CBCL - CHILDHOOD BEHAVIOR CHECKLIST | 43 | 1 | 0 | 0 |
| DAS - DIFFERENTIAL ABILITY SCALES | 43 | 1 | 0 | 0 |
| M-CHAT | 43 | 1 | 0 | 0 |
| NEPSY-II | 43 | 1 | 0 | 0 |
| PEABODY PICTURE VOCABULARY TEST | 43 | 1 | 0 | 0 |
| PEDI - PEDIATRIC EVALUATION OF DISABILITY INVENTORY | 43 | 1 | 0 | 0 |
| REEL-2 - RECEPTIVE EXPRESSIVE EMERGENT LANGUAGE TEST THIRD EDITION | 43 | 1 | 0 | 0 |
| RESCA-E - RECEPTIVE, EXPRESSIVE, AND SOCIAL COMMUNICATION ASSESSMENT | 43 | 1 | 0 | 0 |
| WECHSLER INTELLIGENCE SCALE FOR CHILDREN | 43 | 1 | 0 | 0 |
| WIAT - WECHSLER INDIVIDUAL ACHIEVEMENT TEST | 43 | 1 | 0 | 0 |
| WJ IV - WOODCOCK-JOHNSON TESTS OF COGNITIVE ABILITIES | 43 | 1 | 0 | 0 |
| WOODCOCK-JOHNSON IV | 43 | 1 | 0 | 0 |
| GROSS MOTOR FUNCTION CLASSIFICATION SYSTEM FOR CEREBRAL PALSY | 43 | 0 | 1 | 0 |

**Table 1.B** Peabody Development Motor Scale (PDMS) 2^nd^ Edition frequency

| **PDMS-2 Domain** | **No records** | **1 record** | **2 records** | **3 or more records** |
| --- | --- | --- | --- | --- |
| GRASPING STANDARD SCORE | 9 | 7 | 2 | 1 |
| LOCOMOTION STANDARD SCORE | 13 | 5 | 1 | 0 |
| VISUAL MOTOR INTEGRATION STANDARD SCORE | 12 | 5 | 2 | 0 |
| GRASPING PERCENTILE | 14 | 4 | 1 | 0 |
| GROSS MOTOR QUOTIENT | 15 | 4 | 0 | 0 |
| STATIONARY STANDARD SCORE | 13 | 4 | 2 | 0 |
| LOCOMOTION PERCENTILE | 14 | 3 | 2 | 0 |
| OBJECT MANIPULATION STANDARD SCORE | 15 | 3 | 1 | 0 |
| VISUAL MOTOR PERCENTILE | 16 | 3 | 0 | 0 |
| VISUAL MOTOR STANDARD SCORE | 15 | 3 | 0 | 1 |
| FINE MOTOR QUOTIENT PERCENTILE | 16 | 2 | 1 | 0 |
| FINE MOTOR QUOTIENT STANDARD SCORE | 16 | 2 | 1 | 0 |
| GRASPING AGE EQUIVALENT | 16 | 2 | 0 | 1 |
| OBJECT MANIPULATION PERCENTILE | 16 | 2 | 1 | 0 |
| STATIONARY PERCENTILE | 15 | 2 | 2 | 0 |
| VISUAL MOTOR INTEGRATION PERCENTILE | 16 | 2 | 1 | 0 |
| FINE MOTOR PERCENTILE | 18 | 1 | 0 | 0 |
| FMQ STANDARD SCORE | 18 | 1 | 0 | 0 |
| GRASPING LEVEL AGE EQUIVALENT | 18 | 1 | 0 | 0 |
| GROSS MOTOR QUOTIENT PERCENTILE | 18 | 1 | 0 | 0 |
| LOCOMOTION AGE EQUIVALENT | 16 | 1 | 2 | 0 |
| OBJECT MANIPULATION AGE EQUIVALENT | 18 | 1 | 0 | 0 |
| REFLEXES AGE EQUIVALENT | 18 | 1 | 0 | 0 |
| REFLEXES PERCENTILE | 18 | 1 | 0 | 0 |
| REFLEXES PERCENTILE RANKING | 18 | 1 | 0 | 0 |
| REFLEXES STANDARD SCORE | 18 | 1 | 0 | 0 |
| STATIONARY SKILLS AGE EQUIVALENT | 18 | 1 | 0 | 0 |
| STATIONARY SKILLS PERCENTILE | 18 | 1 | 0 | 0 |
| STATIONARY SKILLS STANDARD SCORE | 18 | 1 | 0 | 0 |
| TOTAL LANGUAGE SCORE | 18 | 1 | 0 | 0 |
| VISUAL FINE MOTOR AGE EQUIVALENT | 18 | 1 | 0 | 0 |
| VISUAL MOTOR AGE EQUIVALENT | 17 | 1 | 0 | 1 |
| VISUAL MOTOR INTEGRATION AGE EQUIVALENT | 18 | 1 | 0 | 0 |
| VISUAL MOTOR LEVEL AGE EQUIVALENT | 18 | 1 | 0 | 0 |
| VMI STANDARD SCORE | 18 | 1 | 0 | 0 |
| GRASPING PERCENTILE RANKING | 18 | 0 | 0 | 1 |
| GROSS MOTOR QUOTIENT STANDARD SCORE | 18 | 0 | 1 | 0 |
| LOCOMOTION PERCENTILE RANKING | 18 | 0 | 1 | 0 |
| STATIONARY AGE EQUIVALENT | 17 | 0 | 2 | 0 |
| STATIONARY PERCENTILE RANKING | 18 | 0 | 1 | 0 |
| VISUAL MOTOR PERCENTILE RANKING | 18 | 0 | 0 | 1 |

**Table 2.** Growth parameter data availability


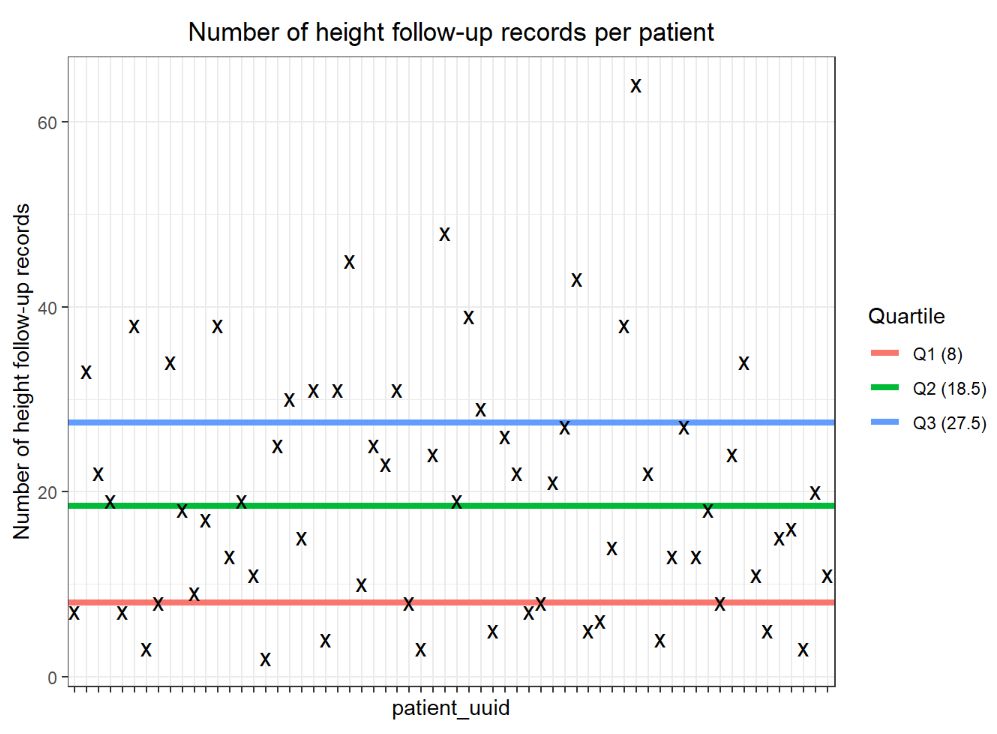


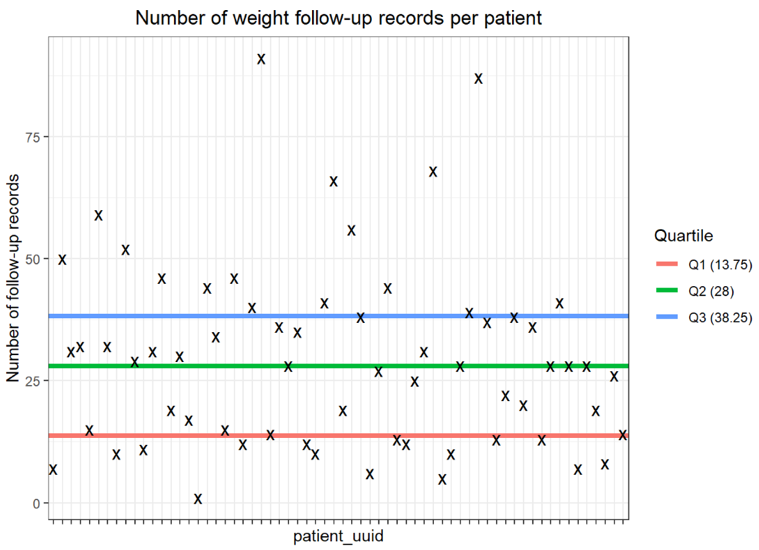


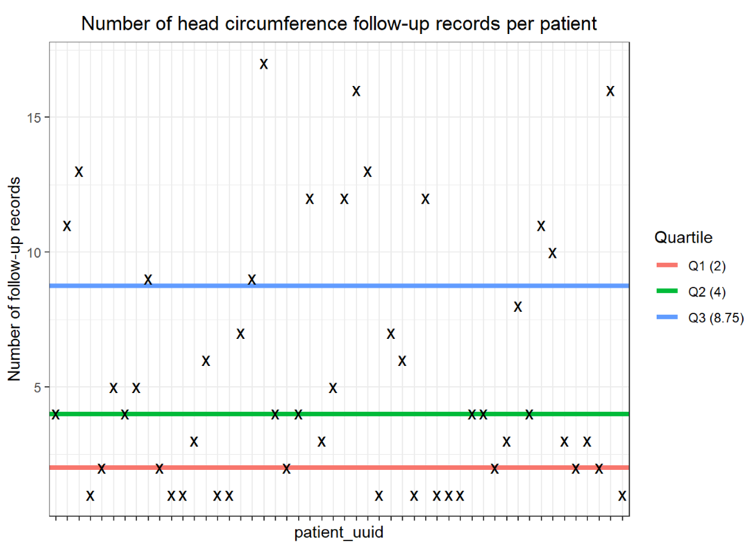


**Table 3.** Developmental skills availability

**Table 3.A.** Total records in each domain

| **Domain** | **Freq** |
| --- | --- |
| Academic Performance | 74 |
| Fine Motor Development | 158 |
| Gross Motor Development | 567 |
| Language Development | 420 |

**Table 3.B.** Academic domain frequencies

| **Domain Skill** | **N Patients** | **Median Age of Ability** | **N Patients with Ability** | **N Multiple Ability Records** | **Median Age of Inability** | **N Patients**  **with Inability** | **N Multiple Inability Records** |
| --- | --- | --- | --- | --- | --- | --- | --- |
| Ability to identify colours | 17 | 4.245038 | 12 | 1 | 3.195072 | 6 | 0 |
| Ability to recognize letters of the alphabet | 15 | 5.488022 | 12 | 0 | 5.37577 | 4 | 0 |
| Ability to write | 8 | 5.69473 | 6 | 0 | 2.195756 | 3 | 0 |
| Ability to count up to 10 | 8 | 5.117043 | 6 | 0 | 6.205339 | 2 | 0 |
| Ability to recognize written words | 5 | 11.82752 | 5 | 0 | *NA* | 0 | 0 |
| Ability to recognize numbers | 5 | 4.339493 | 3 | 0 | 4.276523 | 2 | 0 |
| Ability to read | 4 | 15.62765 | 3 | 0 | 4.049281 | 1 | 0 |
| Ability to recognize symbols | 4 | 5.125257 | 4 | 0 | *NA* | 0 | 0 |
| Ability to understand and use numbers | 4 | 8.246407 | 4 | 0 | *NA* | 0 | 0 |

**Table 3.C.** Gross motor domain frequencies

| **Domain Skill** | **N Patients** | **Median Age**  **of Ability** | **N Patients**  **with Ability** | **N Multiple**  **Ability Records** | **Median Age**  **of Inability** | **N Patients**  **with Inability** | **N Multiple**  **Inability Records** |
| --- | --- | --- | --- | --- | --- | --- | --- |
| Ability to walk | 64 | 1.831622 | 62 | 0 | 1.341547 | 29 | 0 |
| Ability to crawl | 53 | 1.015743 | 49 | 0 | 0.966461 | 15 | 0 |
| Ability to sit | 48 | 0.71321 | 44 | 0 | 0.503765 | 9 | 0 |
| Ability to run | 34 | 2.799452 | 24 | 0 | 3.127995 | 18 | 0 |
| Ability to pull to stand | 34 | 1.058179 | 28 | 0 | 0.966461 | 13 | 0 |
| Ability to stand | 31 | 1.382615 | 25 | 0 | 1.368925 | 6 | 0 |
| Ability to roll | 29 | 0.501027 | 27 | 0 | 0.5859 | 3 | 0 |
| Ability to sit unsupported | 29 | 0.813142 | 28 | 0 | 0.709103 | 8 | 0 |
| Ability to jump | 28 | 4.66256 | 18 | 0 | 4.013689 | 17 | 0 |
| Ability to cruise | 24 | 1.345654 | 20 | 0 | 1.218344 | 8 | 0 |
| Ability to walk with assistance | 20 | 1.941136 | 19 | 0 | 1.319644 | 3 | 0 |
| Ability to control head posture | 17 | 0.243669 | 15 | 0 | 1.054073 | 3 | 0 |
| Ability to roll (front-to-back) | 11 | 0.50924 | 11 | 0 | 0.334018 | 1 | 0 |
| Ability to throw | 11 | 3.268994 | 9 | 0 | 2.684463 | 2 | 0 |
| Ability to stand alone | 10 | 1.255305 | 2 | 0 | 1.190965 | 8 | 0 |
| Ability to roll (back-to-front) | 9 | 0.501027 | 8 | 0 | 0.784394 | 2 | 0 |
| Ability to squat | 6 | 2.113621 | 5 | 0 | 1.28679 | 1 | 0 |
| Ability to belly crawl | 4 | 1.259411 | 4 | 0 | 0.887064 | 1 | 0 |
| Ability to scoot | 3 | 0.999316 | 3 | 0 | 0.887064 | 1 | 0 |
| Ability to stand on one leg | 3 | 7.682409 | 2 | 0 | 3.356605 | 1 | 0 |
| Ability to skip | 3 | 8.213552 | 3 | 0 | *NA* | 0 | 0 |
| Ability to stand from sitting | 3 | 1.494867 | 2 | 0 | 2.338125 | 1 | 0 |
| Ability to kneel | 2 | 2.095825 | 2 | 0 | 1.319644 | 1 | 0 |
| Ability to crouch | 1 | *NA* | 0 | 0 | 1.226557 | 1 | 0 |
| Ability to walk with maximal assistance | 1 | 1.467488 | 1 | 0 | *NA* | 0 | 0 |
| Developmental regression | 0 | *NA* | 0 | 0 | *NA* | 0 | 0 |

**Table 3.D.** Fine motor domain frequencies

| **Domain Skill** | **N Patients** | **Median Age**  **of Ability** | **N Patients**  **with Ability** | **N Multiple**  **Ability Records** | **Median Age**  **of Inability** | **N Patients**  **with Inability** | **N Multiple**  **Inability Records** |
| --- | --- | --- | --- | --- | --- | --- | --- |
| Ability to use pincer grasp | 38 | 1.468857 | 26 | 1 | 1.223819 | 17 | 0 |
| Ability to reach | 31 | 1.155373 | 31 | 1 | 1.054073 | 1 | 0 |
| Ability to perform hand functions | 31 | 1.418207 | 31 | 1 | 1.054073 | 1 | 0 |
| Ability to grasp | 27 | 0.850103 | 26 | 0 | 0.856947 | 2 | 0 |
| Ability to write | 9 | 4.5859 | 6 | 0 | 4.071184 | 3 | 0 |
| Ability to use palmar grasp | 9 | 1.984942 | 8 | 0 | 3.066393 | 1 | 0 |
| Ability to use tripod grip | 1 | 1.330596 | 1 | 0 | *NA* | 0 | 0 |
| Developmental regression | 0 | *NA* | 0 | 0 | *NA* | 0 | 0 |

**Table 3.E.** Language frequencies

| **Domain Skill** | **N Patients** | **Median Age**  **of Ability** | **N Patients**  **with Ability** | **N Multiple**  **Ability Records** | **Median Age**  **of Inability** | **N Patients**  **with Inability** | **N Multiple**  **Inability Records** |
| --- | --- | --- | --- | --- | --- | --- | --- |
| Ability to use at least one word | 53 | 1.763176 | 44 | 1 | 1.404517 | 27 | 3 |
| Ability to use sign language | 41 | 2.587269 | 41 | 0 | 1.333333 | 1 | 0 |
| Ability to babble | 40 | 1.206023 | 32 | 1 | 1.189596 | 16 | 0 |
| Ability to use verbal communication | 36 | 7.679671 | 7 | 0 | 2.595483 | 31 | 0 |
| Ability to vocalize | 32 | 1.208761 | 32 | 3 | 1.578371 | 2 | 0 |
| Ability to use short phrase | 31 | 3.251198 | 20 | 0 | 2.475017 | 14 | 0 |
| Ability to use non-verbal communication | 28 | 2.505133 | 27 | 0 | 1.690623 | 4 | 0 |
| Ability to recognize spoken words | 23 | 1.672827 | 23 | 1 | 1.138946 | 3 | 0 |
| Ability to follow commands | 16 | 2.951403 | 15 | 0 | 2.190281 | 5 | 0 |
| Ability to speak | 10 | 4.709103 | 5 | 0 | 3.559206 | 5 | 0 |
| Ability to use sentences | 9 | 7.151266 | 5 | 0 | 4.199863 | 5 | 0 |
| Ability to use augmentative and alternative communication | 9 | 4.629706 | 8 | 0 | 2.951403 | 1 | 0 |
| Ability to use word approximations | 8 | 2.176591 | 6 | 0 | 2.043806 | 2 | 0 |
| Ability to communicate | 6 | 4.621492 | 5 | 0 | 5.067762 | 1 | 0 |
| Ability to speak intelligibly | 5 | 5.511294 | 1 | 0 | 3.262149 | 4 | 0 |
| Ability to speak fluently | 3 | 6.316222 | 1 | 0 | 3.472964 | 2 | 0 |
| Ability to use language | 2 | 3.208761 | 1 | 0 | 3.011636 | 1 | 0 |
| Ability to speak own name | 2 | 4.010951 | 1 | 0 | 3.356605 | 1 | 0 |
| Developmental regression | 0 | *NA* | 0 | 0 | *NA* | 0 | 0 |
